# Supplementary material for: Mechano Chemical Compatibilization of Polyethylene with Graphite by Means of a Suitable Ester
Source: Polymers (Basel). 2023 Jun 21;15(13):2770. doi: 10.3390/polym15132770 (PMC10346664; doi:10.3390/polym15132770)
Supplement: Supplementary file 1 [file polymers-15-02770-s001.zip › polymers-2449687-supplementary.pdf]

Supplementary Materials

Mechano Chemical Compatibilization of Polyethylene with Graphite by Means of a Suitable Ester

Simona Russo [1], Maria Rosaria Acocella [1,*], Annaluisa Mariconda [2,*], Valentina Volpe [3], Roberto Pantani [3] and Pasquale Longo [1]

[1] Department of Chemistry and Biology, University of Salerno, Via Giovanni Paolo II, 132, 84084 Fisciano, Italy; sirusso@unisa.it (S.R.); plongo@unisa.it (P.L.)
[2] Department of Science, University of Basilicata, Viale dell'Ateneo Lucano 10, 85100 Potenza, Italy
[3] Department of Industrial Engineering, University of Salerno, Via Giovanni Paolo II, 132, 84084 Fisciano, Italy; vavolpe@unisa.it (V.V.); rpantani@unisa.it (R.P.)
* Correspondence: macocella@unisa.it (M.R.A.); annaluisa.mariconda@unibas.it (A.M.); Tel.: +39-089-968105 (M.R.A.); Tel.: +39-0971-205932 (A.M.)

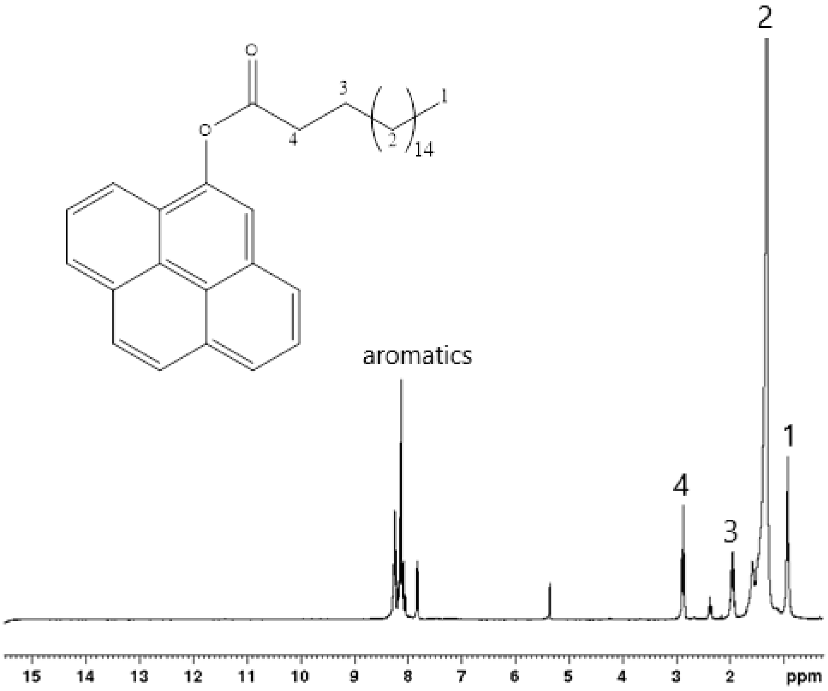

Figure S1. ^{1}H NMR spectrum of pyren-1-yl-stearate (P1S)

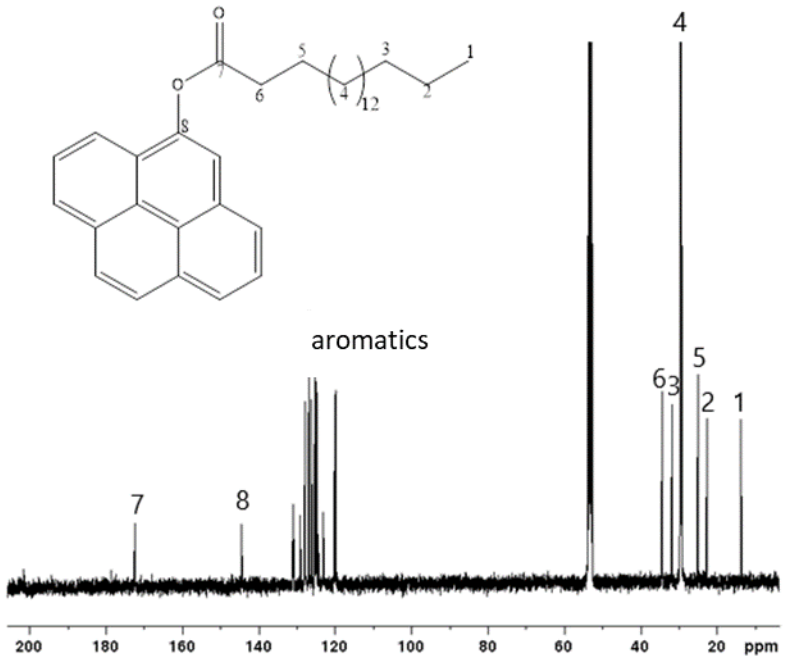

Figure S2. ^{13}C NMR spectrum of pyren-1-yl-stearate (P1S)
